#  Evaluation of Manual and Two-Rotary Niti Retreatment Systems in Removing Gutta-Percha Obturated with Two Root Canal Sealers

**DOI:** 10.5402/2012/208241

**Published:** 2012-09-10

**Authors:** Athikesavan Jayasenthil, Emmanuel Solomon Sathish, Prashanth Prakash

**Affiliations:** ^1^Department of Conservative Dentistry and Endodontics, Tagore Dental College and Hospital, Rathinamangalam, Vandalur, Chennai 600048, India; ^2^Department of Conservative Dentistry and Endodontics, Rajah Muthiah Dental College and Hospital, Annamalai University, Annamalai Nagar 608002, India

## Abstract

*Objective*. The objective of this study was to evaluate the efficacy of two retreatment NiTi systems (protaper universal retreatment files, R-Endo), when compared to manual technique in removing Gutta-percha obturated with two sealers. *Study Design*. Sixty extracted single-rooted premolars were instrumented with Protaper rotary files till F3. The specimens were divided into six groups. Groups 1, 2, 3 were obturated with Gutta-percha and zinc oxide eugenol and Groups 4, 5, 6 were obturated with Gutta-percha and AH-plus. The retreatment was carried out in groups 1 and 4 with H-files and GGdrills, groups 2 and 5 with R-endo retreatment files and groups 3 and 6 with Protaper retreatment files. The roots were sectioned and evaluated under optical stereomicroscope. Statistical analysis was performed with one-way ANOVA and Newman-Keul's test at *P* < 0.05. *Results*. The manual technique resulted in cleaner canal walls when compared with both rotary retreatment systems. *Conclusion*. NiTi rotary retreatment files can be used to remove the filling material quickly, but it should be followed by hand instruments to obtain better canal wall cleanliness.

## 1. Introduction

A growing interest in endodontic retreatment has been seen recently, caused by an increasing demand to preserve teeth, including those cases where endodontic therapy had failed [1]. Endodontic failure may occur in cases where there are persistent bacteria present due to insufficient cleaning or inadequate obturation [2]. Endodontic retreatment can be carried out either by nonsurgical or surgical procedure. Ideally all the filling materials should be removed from the canal to gain access to microorganisms and remnant tissues [3]. The commonly encountered root-canal filling material requiring removal is gutta-percha [4]. Gutta-percha is usually removed with Hedstrom files alone or in combination with Gates Glidden drills (GGdrills) with or without solvents [3]. Other techniques proposed include heated instruments, rotary files, ultrasonic instruments, and lasers [5–7].

Various nickel titanium rotary instruments have been developed to facilitate cleaning and shaping of root canals. They had advantages such as maintenance of canal shape, no canal deformation, and shorter working time [8, 9]. Rotary NiTi files have also been proposed for the removal of gutta-percha and several studies also have been done to check their efficacy and safety. Different rotary systems such as Profile, Quantec, GT rotary, K3, Protaper and Race have been evaluated for the root filling removal [5, 9–11]. Recently two rotary NiTi retreatment systems have been introduced; they are Protaper universal retreatment files and R-Endo files. They are specifically designed for retreatment.

Protaper universal retreatment system has 3 grey coloured files D1, D2, and D3. The D1 has an active cutting tip and has a length of 16 mm and tip of 0.30 mm with 0.09% taper. The D1 is used for initial penetration. The D2 used in middle third has a length of 18 mm and tip of 0.25 mm with 0.08% taper. The D3 is used in apical third has length of 22 mm and tip of 0.20 mm with 0.07% taper.

The R-Endo system consists of 4 rotary files and one hand file (Re, R1, R2, R3, and Rm). Rm is only for initial penetration because all rotary files are nonend cutting. Re is used in initial bulk removal of filling material with 0.12% taper and 15 mm length. R1 is used in coronal third with 0.08% taper 15 mm length. R2 is used in middle third with 0.06% taper and 19 mm. R3 is used in apical third with 0.04% taper and 23 mm length with diameter. The aim of this in vitro study was to compare the efficacy of manual technique, Protaper universal rotary retreatment system and R-Endo retreatment system in removing gutta-percha and also the influence of type of sealer on presence of filling debris in the reinstrumented canals.

## 2. Material and Methodology

### 2.1. Specimen Selection

Sixty mandibular single-rooted premolars with fully formed apex were included in the study. All teeth were stored in sterile water. Teeth with root caries, previous root canal treatment, and immature apex were excluded from this study.

### 2.2. Initial Endodontic Treatment

The crowns were flattened; so that root canals showed a standardized working length of 18 mm. Accesses opening were made in all specimens. A 10-K file was placed into the canal until it was visible at the apical foramen and the working length was established at 1 mm short of that length. Endodontic treatment was performed using Protaper Universal NiTi rotary instruments. Canals were enlarged up to F3 at working length. During instrumentation all canals were irrigated between each instrument change with 2.5 mL of 5.25% NaOCl. A final flush was performed with 5 mL of 17% EDTA for 30 seconds followed by a rinse with 5 mL of saline.

The specimens were divided into 6 groups of 10 teeth each. After drying the canals with paper points the obturation was carried out by cold lateral condensation. Groups 1, 2, and 3 were obturated with gutta-percha and zinc oxide eugenol sealer and in groups 4, 5, and 6 were obturated with gutta-percha and AH plus sealer. The quality and extent of obturation were confirmed radiographically. The access cavities were sealed with Cavit-G and stored at 100% humidity for 2 weeks.

### 2.3. Endodontic Retreatment

In groups 1 and 3, the retreatment was carried out with GG-drills and H-files. At first GG- drill size 3 was used at the coronal 3rd and 0.1 mL of RC solve solution is placed for 3 minutes and then GG drill 2 was used up to middle third and H-files is used in crown down manner to end at size 30 at the apex. First Hedstrom files of sizes 30–15 were used in descending order to the working length using a circumferential filling motion. Once the working length had been reached with a size 15 file, sizes 20, 25, and 30 were used at the working length.

In groups 2 and 5, the retreatment was carried out with R-Endo retreatment files. First Rm hand file (K-File) was used with 1/4 turn pressure directed towards the apex to create a pathway; thus, allowing the centering and the alignment of the next instrument. Re NiTi rotary file was used 1 to 3 mm beyond the pulp chamber floor with circumferential filing. Again, 0.1 mL of RC solve was deposited into the reservoir created for 3 min. R1 NiTi rotary file was used to penetrate from the coronal third to the beginning of the middle third through repeated apically directed pushing actions. R2 NiTi rotary file was used from the middle third to the beginning of the apical third. R3 NiTi rotary file was used at the working length with circumferential filing action.

In groups 3 and 6, the retreatment was carried out with Protaper universal retreatment files. The D1 Protaper file was used to remove the filling material from the cervical third of the root canal and 0.1 mL of RC solve was deposited for 3 min into the reservoir created. A D2 Protaper file was used in the coronal two thirds of the root canal. The D3 Protaper file was used with light apical pulses of pressure until the working length was reached. All the groups retreatment was done until no further filling material could be removed. Both the rotary retreatment systems were used in endodontic micromotor (X-Smart) at 300 rpm in crown down manner. The time taken for the retreatment procedure was calculated using a stop watch.

### 2.4. Analysis

The roots were grooved longitudinally in buccolingual direction with a diamond disk and split into two halves with a chisel. The sectioned specimens were observed under optical stereo microscope at 8x magnification and images were captured. The images were transferred to ImageJ image analysing software and canal area of each thirds and amount of debris present in each third were calculated. A grading system was used to score amount of debris and remaining filling material for the coronal, middle, and apical portions separately [12].

 0: None-to-slight presence (0%–25%) of residual debris.
 1: Presence of 25% to 50% of residual debris.
 2: Moderate presence (50%–75%) of residual debris.
 3: The entire or almost the entire surface (75%–100%).

### 2.5. Statistical Analysis

Statistical analysis was performed using Graph Pad Prism software. One-way ANOVA was performed to calculate the mean and standard deviation. Newman-Keuls test was done for pair-wise comparison between groups at each third of the canal separately at *P* < 0.05.

## 3. Results

The mean and standard deviation of remaining obturating material of all groups were reported in (Table 1). There was of no statistical difference in the coronal and middle thirds of the canal between the groups.

In case of apical third of the canal, groups reinstrumented by manual technique (1 and 4) showed significant when compared to groups (reinstrumented with protaper retreatment files (3 and 6). In total the amount of remaining debris was less in groups treated with manual technique than rotary retreatment groups.

In case of time taken for the retreatment procedure, both the rotary systems took less time (Table 2) for removal of gutta-percha than manual technique.

## 4. Discussion

The preferred treatment of failing endodontic cases is nonsurgical retreatment. The procedure requires the removal of the original root canal filling, further cleaning, and refilling [1]. Removal of sealer and gutta-percha from inadequately prepared and filled root canal systems is essential in root canal retreatment because it is likely to uncover remaining necrotic tissue or bacteria that may be responsible for periapical inflammation and posttreatment disease [13].

It was not possible to remove all the traces of gutta-percha and sealer from the root canal walls with any of the techniques, which has also been reported in other studies [14–16]. Furthermore, in this study the manual technique for retreatment with Hedstrom files and Gates Glidden drills was found to render cleaner canals compared to Protaper and R-Endo group retreatment files.

The teeth were flattened coronally and the working length of each root canal was standardized at 18 mm so that varying lengths could not exert influence on the results, which was followed in previous study [5].

In current study orange oil was used as a solvent which have been reported effective and less cytotoxic than eucalyptol, xylol, chloroform, and halothane [17]. In all the groups' residual filling material was found to be more in apical third than middle and coronal third. This may be due to accumulation of more debris apically regardless of the protocol or material used [16, 18].

Retreatment was performed in significantly less time using two rotary retreatment systems than the manual technique which was consistent with the previous results [10, 15, 19–22]. Both the time required and remaining filling material were not statistically significant between both sealers with all the techniques.

The evaluation of remaining filling material was performed by calculating the percentage of debris in each third of the canal in relation to the canal area of each third. This method was supported and performed in previous studies to evaluate the amount of remaining filling material [10, 12]. In this method the remaining debris is visualized three dimensionally and the error is minimized compared to radiographic technique where only the two-dimensional view is possible.

The manual retreatment technique resulted in cleaner canal walls in the apical third compared with the engine-driven NiTi rotary systems. This may be due to the master apical file size selected for retreatment in this study. The master apical file size used for the manual technique was the same as the file selected for the root canal preparation, that is, no. 30, where tip size was 20 and 25 for Protaper and R-Endo retreatment files. 

Within the limitations of this study the results showed that NiTi rotary instrument and hand instruments both left remnants on the root canal walls; mostly in the apical third of the canal. NiTi rotary retreatment files can be used to remove the filling material quickly but it should be followed by hand instruments to obtain better canal wall cleanliness especially in the apical third of the root canal.

## Figures and Tables

**Table 1:** Mean scores (SD) of canal wall cleanliness evaluated by means of OSM analysis for the different groups at the coronal, middle, and apical portions.

Groups	Coronal 3rd		Middle 3rd		Apical 3rd	
	Mean	SD	Mean	SD	Mean	SD
Group 1	0.4	0.51	0.7	0.67	1	0.66
Group 2	0.7	0.48	1.2	0.63	1.6	0.63
Group 3	0.4	0.51	1.1	0.56	1.8	0.69
Group 4	0.5	0.52	0.8	0.63	1	0.66
Group 5	0.8	0.42	1.3	0.67	1.6	0.63
Group 6	0.6	0.51	1.2	0.63	1.8	0.69

**Table 2:** Mean time necessary for retreatment of different groups.

Groups	Time required (mean) min
1	8.36
2	5.32
3	5.08
4	9.08
5	5.58
6	5.13
